# Changes in sport and physical activity participation for adolescent females: a longitudinal study

**DOI:** 10.1186/s12889-016-3203-x

**Published:** 2016-07-08

**Authors:** Rochelle M. Eime, Jack T. Harvey, Neroli A. Sawyer, Melinda J. Craike, Caroline M. Symons, Warren R. Payne

**Affiliations:** Institute of Sport, Exercise and Active Living, Victoria University, Melbourne, Australia; School of Health Sciences and Psychology, Federation University, Ballarat, Australia

**Keywords:** Sport, Physical activity, Adolescent females, Setting, Health

## Abstract

**Background:**

Participation in sport and physical activity is reported to decline during adolescence, particularly for females. However we do not have a clear understanding of changes in the context (i.e., modes and settings) of participation throughout adolescence. This study investigated longitudinal changes in physical activity participation and the specific modes and settings of physical activity, together with cross-sectional comparisons, for two age cohorts of female adolescents.

**Methods:**

Survey of 729 adolescent girls (489 recruited in Year 7 and 243 in Year 11). Participation in eight different modes/settings was reported. PA was measured using 24-h recall diary and metabolic equivalent weighted energy expenditure (MET-min) in Leisure Time Moderate and Vigorous Physical Activity (LTMVPA) on the previous day was calculated.

**Results:**

There were no significant changes in duration or total MET-min of LTMVPA on previous day. However, there were significant changes in the modes/settings of participation across time. Participation in school physical education rose during early adolescence before decreasing significantly, and participation in competitive sport and club sport significantly decreased over time; however there were increases in non-competitive forms of physical activity.

**Conclusions:**

Overall levels of physical activity did not significantly decrease over adolescence, which is positive for physical health. However, the transition from structured sport to non-organised physical activity may effect social and psychological health, which needs to be further examined.

## Background

There are a range of physical and mental health benefits associated with regular participation in physical activity (PA) [1]. However, many people are not sufficiently physically active to gain these health benefits [2]. More specifically, many adolescents do not participate at levels that meet the recommendations for health [3–5]. Female adolescents are consistently reported as being less physically active than their male peers [4, 6]. Furthermore, there is evidence that overall levels of PA decrease during adolescence [4, 5, 7]. This is also true for specific contexts of participation, such as sport [8]. A clear understanding of the contexts of participation and how these change throughout adolescence is required to inform strategies for improving participation.

There are inconsistent findings regarding the changes in PA participation throughout adolescence. Overall, for adolescent girls the annual percent decline in PA in a cross-sectional sample was approximatly 4 % or -1.76 min of Moderate and Vigorous Physical Activity (MVPA) per day [7]. However, another cross-sectional sample in Australia found no significant difference between the proportions of Year 7 and Year 11 girls exceeding or meeting the recommended level of MVPA [3]. Longitudinal research has demonstrated that despite a decline in PA participation into adolescence for the majority of people, some girls (24 %) maintain a stable level of participation throughout childhood and adolescence [9]. More specifically, within the school setting, longitudinal research has indicated there are significant decreases in recess and lunchtime MVPA [10].

The context of leisure-time PA has three aspects which have been termed *mode*, *setting* and *type* [3]. Four *modes* of participation were distinguished: team sport (e.g. netball), individual sport (e.g. tennis), organized but non-competitive PA (e.g. aerobics), and non-organised PA (e.g. walking). The three main *settings* for PA in adolescents are: school; club or leisure center; and neighborhood settings such as home, street or park. The *type* of PA is defined by the many specific sports and forms of leisure-time PA such as tennis, swimming and walking [3]. Other researchers have used the terms *structured* and *unstructured* to characterise different modes of participation [11]. PA participation is complex, as it is common for multiple modes and/or settings to be involved when partcipating in a particular type of PA [3].

Cross-sectional research has shown that there are differences between younger and older female adolescents in their modes of participation in PA [3]. The younger cohort was more likely to participate in team sport and individual sport, whereas the older cohort was more likely to participate in organised but non-competitive PA [3]. A recent study which investigated social influence and PA in structured and unstructured settings, found that particular types of social influences were specific to different modes of participation [11]. This study defined three types of influence: conformity (“It’s cool to go snowboarding with my friends”), compliance (“Coach convinced me to join wrestling”) and obedience (“Parents told me to go play”). The findings suggested that family-compliance and peer-compliance influenced participation in structured PA settings, whereas peer compliance and conformity were the most frequently reported social influences in unstructured settings.

Although there is some knowledge about participation within different modes of PA, little is known about participation across different settings. This is partly explained by the measures of PA that have been used in previous research. For example, studies that utilise objective measurements are unable to account for the specific setting of PA, and many PA recall measurements do not assess the setting of participation [4, 7, 10]. Notwithstanding this, some research has focused on one particular setting, often the school setting [10].

There is evidence that participation in PA in certain contexts predicts future overall levels of PA. For female children and adolescents, playing sport outside school has been found to be positively associated with being persistently active during the transition from adolescence to adulthood [12]. Similarly, non-membership in a sports club has been shown to be a strong predictor of ‘no sport’ participation and also for being inactive in adolescence [8].

A limitation of current research is that information about how participation in PA changes through the adolescent period is scarce. Although there is some information on changes in overall levels of PA and how some determinants change over time, there is limited research on changes in mode or setting of participation. To address this shortcoming, the present study investigated longitudinal changes in PA participation, and the specific modes and settings of PA, together with cross-sectional comparisons, for two age cohorts of female adolescents.

## Methods

Schools in metropolitan, regional and rural areas of Victoria, Australia, were randomly selected and invited to participate. The postcodes of schools were used to assign a value of the Australian Bureau of Statistics (ABS) SEIFA (Socio-economic Indices for Areas) Index of Relative Socio-economic Advantage and Disadvantage (IRSAD) [13] to each, and the distribution of schools was checked to ensure they were representative of the broader IRSAD distribution in Victoria.

Sample targets were 600 metropolitan and 300 non-metropolitan participants for each of Year 7 and Year 11 cohorts of female students. In terms of statistical power, under the assumption of moderate prevalences (between 30 % and 70 %), and with negligible cluster effects, a sample of size 900 was determined to be sufficient to detect and odds ratio of 1.6 when comparing two categories such as presence/absence of sufficient PA to incur a health benefit and presence/absence of some explanatory factor such as self-determination or adequate access to facilities. Based on an estimated recruitment uptake of 60 %, and estimated retention of 75 % between waves 1 and 3, the initial contact targets for each of Year 7 and Year 11 cohorts were 1333 metropolitan and 667 non-metropolitan. Based on information from school websites and key informants, we estimated average school populations of 800 (metropolitan) and 500 (non-metropolitan). Assuming equal numbers of boys and girls, and equal numbers in each of the 6 year levels, this led to targets of 20 metropolitan schools and 15 non-metropolitan schools. A total of 17 schools in the metropolitan area (34 % of 50 contacted) and 14 schools in rural and regional areas (88 % of 16 contacted) participated in the study. Ethical approval was gained from the Human Research Ethics Committee of Federation University Australia, the Victorian Department of Education and the Victorian Catholic Education Office.

Development of a survey questionnaire was informed by a prior qualitative study in which girls in sixteen of the recruited schools (eight metropolitan and eight non-metropolitan) participated in focus groups [14, 15]. A pilot test was also conducted involving 71 respondents in a convenience sample of three schools. Based on the findings of this pilot study, minor changes were made to the content of the questionnaire and the order of questions was revised.

There were three waves of data collection. During the southern hemisphere Autumn (April 2008), all female students in years 7 and 11 of participating schools were invited (by the physical education (PE) coordinator or a researcher) to participate in the study, and plain language information statements, parental and respondent consent forms were distributed. Students who returned both self and parent completed consent forms within the stipulated time completed the baseline questionnaire, usually during school class time. Subsequently questionnaires were distributed in April 2009 and April 2010. Because the students recruited in Year 11 generally finished school in December 2009, the 2009 questionnaire included a request for contact details (phone and/or email address) for the distribution of the 2010 questionnaire.

The potential for self-selection or response bias was addressed by the use of display materials and briefing notes for teachers which stressed the importance placed by the researchers on the experiences and opinions of less physically active girls, and by offering incentive prizes. Furthermore, the critical role of well-motivated school staff in promoting recruitment and adherence of participants was also recognized. Consequently, small incentive prizes were offered in the form of a gift voucher awarded to a randomly chosen participant in each school, a gift voucher to the coordinating teacher in each school and a voucher to each school for the purpose of equipment purchase.

The variables used in this analysis are listed in Table 1, in two groups: modes and settings of PA [3] and PA levels. The modes and settings are categorical variables (Yes/No dichotomies) and the PA levels are quantitative scales.

**Table 1:** Modes & settings and PA levels: Longitudinal changes within cohorts and differences between cohorts

			Cohort, calendar year and school year level															
			Year 7 (*n* = 328^a^)							Year 11 (*n* = 112^a^)								
	Measure/dichotomy		2007^b^	2008	2009	2010	Statistically significant trend^c^			2007^b^	2008	2009	2010	Statistically significant trend^c^			Statistically significant difference between cohorts^,^	
Characteristic		Variable	Yr 6	Yr 7	Yr 8	Yr 9	*p*-value	Type	Sign	Yr 10	Yr 11	Yr 12	Yr 13	*p*-value	Type	Sign	*p*-value	Sign^d^
MODES & SETTINGS OF PA																		
Physical Education classes	Yes/No	% Yes	82	97	99	96	< .001	Linear Quadratic	+	89	46	34	N/A^e^	< .001	Linear	-	< .001	-
							< .001		+ -					< .001	Quadratic	- +		
Competitive team sports - at school	Yes/No	% Yes	80	65	64	61	< .001	Linear	-	69	60	52	N/A^e^	< .001	Linear	-	.002	-
Competitive team sports - outside school	Yes/No	% Yes	66	63	63	58				67	64	53	47	< .001	Linear	-		
Competitive individual sports - at school	Yes/No	% Yes	49	45	35	35	< .001	Linear	-	45	32	32	N/A^e^	.001	Linear Quadratic	-		
														.034		- +		
Competitive individual sports - outside school	Yes/No	% Yes	41	37	38	33				38	24	20	18	< .001	Linear	-	<.001	-
Organised but non-competitive physical activity e.g. aerobics, weights class, circuit training	Yes/No	% Yes	36	32	43	49	< .001	Linear	+	51	47	51	55				.003	+
Non-organised physical activity e.g. walking, jogging, rollerblading (alone or with friends)	Yes/No	% Yes	70	81	87	85	< .001	Linear Quadratic	+	81	92	92	92	.045	Quadratic	+ -	<.001	+
							.002		+ -									
Sports club member	Yes/No	% Yes		70	69	67					66	54	50	.001	Linear	-	.012	-
PA LEVELS																		
Duration of LTMVPA (min) on previous day (min)	Scale (0-)	Mean		68.6	73.0	74.0					82.2	72.1	62.5					
Total MET-min of LTMVPA on previous day	Scale (0-)	Mean		489.2	523.6	560.4					601.0	533.6	498.4					

^a^For PDPAR-based measures (LTMVPA and MET-min) sample sizes in the three waves were: Year 7 *n* = 214, 215 and 221; Year 11 *n* = 84, 89, 71

^b^At baseline (2008), participants were asked about their modes and settings of PA 1 year earlier (2007)

^c^ *p*-values for each statistically significant longitudinal trend. Signs indicate direction of linear trend and pattern of quadratic curvature superimposed on linear trend

^d^ *p*-values for each statistically significant difference. Signs indicate the direction of difference between Year 7 and Year 11 cohorts

^e^School-based activities were not applicable in Year 13 (post-school)

Respondents were asked whether or not they participated in PA in seven mode/setting combinations: school PE classes; competitive team activities and competitive individual activities both in and out of school; organized non-competitive activities; and non-organized activities. With regard to the question about modes/settings, respondents were also asked to answer with respect to the previous year (i.e. when they were in Year 6 or Year 10) as well as the current year (Year 7 or Year 11). They were also asked whether they were currently a member of a sports club [3].

The level of PA was estimated using a validated 24-h recall diary—PDPAR [16]—modified to focus exclusively on female activities. Estimates of the rate of energy expenditure in metabolic equivalents (METs) were derived from the PDPAR data using the Compendium of Physical Activities [17]. MVPA was defined as any activity with a MET ≥ 3.0. The number of 30-min blocks of LTMVPA and hence the total LTMVPA (min) and total MET-weighted LTMVPA (MET-min) were derived from the responses for leisure-time activities in the PDPAR diary.

Data screening was undertaken prior to the data analyses. Preliminary analyses to compare the baseline characteristics of those who completed all three surveys (‘completers’) and those who did not (‘non-completers’) were conducted using chi-square and independent samples t-tests. For the longitudinal analysis of each quantitative measure or dichotomised characteristic, cases with complete data were analysed using linear mixed models and generalised estimating equations to identify statistically significant differences between the two cohorts and statistically significant longitudinal trends—linear and non-linear—within each cohort. All analyses were conducted using SPSS Version 19.

## Results

The 2008 consent/response rate was 19.6 % (Year 7 24.1 %, Year 11 14.2 %) with retention rates in 2009 and 2010 of 82.4 % (Year 7 83.6 %, Year 11 80.7 %) and 74.0 % (Year 7 81.9 %, Year 11 57.7 %) respectively. Respondents who returned all three survey forms comprised: Year 7 (*n* = 328, 74.5 %; aged 11–13, M ± SD = 12.2 ± 0.5 years at baseline) and Year 11 (*n* = 112, 25.5 %; aged 16–18, 16.2 ± 0.6 years at baseline).

There were some differences between the baseline characteristics of those who completed all three surveys (‘completers’) and those who did not (‘non-completers’), which have been reported and discussed elsewhere [18]. Briefly, there were no significant differences in age, SES (SEIFA IRSAD score of residential postcode), self-reported BMI in either cohort; in the Year 7 cohort completers had a lower self-reported weight at baseline than non-completers. Across both cohorts, completers were significantly more likely than non-completers to report participating in some forms of sport and PA.

For analysis associated with the PDPAR-24, specific data quality checks were undertaken, and participants who left gaps in the diary or who reported inappropriately high intensity levels for sedentary activities such as sleeping and sitting were excluded. Cases with estimated MVPA of more than 256 mins/day (i.e. those who reported more than eight 30-min blocks), which corresponds to the 99th percentile of responses in the 2007 Australian National Children’s Nutrition and Physical Activity Survey (Prof Tim Olds, personal communication, 14 December 2011), were also excluded. The resulting number of participants eligible for inclusion in the analysis of MET-mins and time spent in MVPA in the three waves of the survey were: Year 7 *n* = 214, 215 and 221; Year 11 *n* = 84, 89, 71.

Table 1 summarises the results of the analysis for each outcome variable. With regard to the modes and settings of PA, each variable is the percentage of respondents who participated in a particular mode/setting. The variables relating to PA levels comprise a variety of scales and one dichotomous variable.

Table 1 shows that participation rates ranged widely in the eight modes and settings examined, from around one-third for non-organised PA among the youngest girls to almost 100 % for school PE classes in Years 7–9. Participation in school PE increased sharply in the transition from primary (Year 6) to secondary (Year 7) school and decreased sharply at Year 11. Participation in competitive sports and membership of sports clubs generally decreased over time in both cohorts and between the younger and older cohorts, although not all trends/differences were statistically significant. Conversely, participation rates in non-competitive settings trended upwards in the younger cohort, and were significantly higher in the older cohort than in the younger cohort. The duration of LTMVPA and MET-mins of LTMVPA on the previous day both increased over time in the younger cohort and decreased over time in the older cohort, but not to a statistically significant extent.

## Discussion

The current study extends previous research by prospectively, as well as cross-sectionally, examining the trends in participation in PA as well as changes in contexts of participation in PA for female adolescents.

It is encouraging that neither the average duration of MVPA, or total MET-mins changed significantly over time. That is, PA levels did not significantly decline across adolescence.

There was substantial variation in participation in PA in different modes and settings. Transition into secondary school was associated with increases in school Physical Education (PE), however participation in PE dropped sharply in later adolescence. It has been suggested that one reason for the pattern of increased school PA in early adolescence is that there are more PA options in secondary than in primary or elementary school [6]. The decline in school PA in later adolescence may be explained by a number of factors including changes in life priorities and time demands, shifting towards academic achievement and paid empoyment [14], and a lack of support from families [18, 19] friends, and teachers [15] . More broadly, there are generational social and cultural influences related to role modeling of sport participation for males and females [20], in particular the role of sport as an important definer of masculinity in mass culture [21]. Hence, for young males there is a relatively easy fit with their sporting identity and masculine identity, whilst with young females this relationship is not straightforward and there are greater societal expecations for males to be involved in sport than for females [22]. Within the familial context, children’s participation in sport is influenced by the parents’ own sporting backgrounds and males are more likely than females to participate as adults [19, 20, 23]. Furthermore, the decline may be due to a lack of PA options that adolescent females would favour. For example the competitive nature of sport is not attractive to many adolescent females because they would prefer more options to socialise, they believe that they may not gain a position of a team [15], or they lack the level of competence required to participate in the team [18].

Participation in competitive sports and membership in sports clubs generally decreased over time in both cohorts and between younger and older adolescents. Similarly, in a longitudinal study of people aged 14 and 24, the prevalence of no sport, and non-membership in a sport club increased with age [8]. Although participation in sport via membership of a sports club decreased with age (from 70 % in Year 7 to 50 % in Year 13) in our study, there were increases evident in organised but non-competitive PA (32 % to 55 %), and also in the non-organised PA mode, (81 % to 92 %). Another study of females aged 15–19, 20–24 and 25–29 found that whilst basketball and ‘other sports’ were popular among adolescents, more individual-based activities as well as sports played with their children were popular in the oldest cohort [24]. This change is likely to be due to decreasing capacity or inclination to fit in with the organised structure and time commitments of sport, and the increasing importance of other life priorities and time commitments [14]. However, the increase in non-organised PA indicates that being physically active is still important to older adolescents, which is a positive for health.

Although non-organised PA has health benefits it is important to recognise that participation in organised PA such as sport has unique benefits for adolescents that might not be achieved through non-organised types of PA [25]. Firstly, participation in sport during early adolescence is related to a higher likelihood of being active during late adolescence [26]. Sport participation contributes to overall vigorous PA during late adolescence, when overall PA has been reported to decline [26]. Similarly there is a strong correlation between sports club membership and PA [8]. Several longitudinal studies have shown that sports participation during adolescence is postively associated with adult participation in PA [27–29]. A study of females over 20 years showed that sport participation during adolescence is a better predictor of adults’ involvement in sports than educational level or parental socioeconomic status [27]. In another study, participation in club sport at least once a week was associated with a high level of PA for females in later life [28]. Many adolescents enjoy participation in sport, however many drop out, and therefore PA and sport need to be positioned in the context of the lived experiences of female adolescents using individual and organisational strategies to encourage and support participation [18].

Whilst the current study presents the changes in sport and PA across adolescence, the socioecological determinants associated with these changes are also important. Changes in socioecological factors in relation to this study have been previously reported [18]. In summary, lack of time was more prevalent amongst the Year 11 cohort than the Year 7 cohort, as were perceived importance of life priorities relating to education and study. Furthermore, perceived competence declined over time for the Year 7 cohort. For both cohorts support from friends and familes declined over time, whereas access to facilities increased [18]. Earlier qualitative studies of females from the same schools and in the same age cohorts as the current study investigated levels and determinants of sport and PA and reported some similarities but many differences between the two age cohorts [14, 15]. Both Year 7 and Year 11 cohorts reported that they participated in PA and sport for fun and enjoyed participating with their friends [14, 15]. However the younger cohort reported that support from families and teachers in the form of role models and positive feedback was an important determinant as was competency and single-sex classes/lessons [15]. On the other hand, the older girls reported that educational priorities conflicted with their capacity to participate, and that they were likely to transition from club based sport participation to more unstructured, flexible, but socially isolating, activities [14]. The girls included in the current study reported that family support, via encouragement, praise, watching/supervision, or direct involvement in PA—decreased over time [18]. Strategies are required to promote and maintain family support for adolescent females participating in PA and sport [18, 23], especially because females appear to receive less encouragement to be active than do males [19]. Female participation in sport is impacted by their parents’ participation, and mothers are often less likely to play competitively than fathers which may be related to generational social and cultural influences more broadly [20].

The strengths of the current study include a comprehensive assessment of PA participation and contexts, and the prospective study design, which allowed the patterns of PA to be assessed longitudinally over time in the same cohorts of adolescents. Studies of this population segment are very hard to conduct, particularly in light of the ethical requirement of Australian education authorities to obtain specific ‘opt-in’ parental consent, which is exacerbated by the necessity to communicate with parents only indirectly in writing via the school and the students themselves. The research team was dependent on the efforts of teachers with many competing priorities to facilitate and promote recruitment and retention, amid the complexities of school operations and scheduling, and the competing demands on the time of students, particularly at the senior level. Consequently, limitations to this study include low consent and recruitment rates, particularly among students in Year 11, and moderate retention rates. There was a particularly low retention rate in Year 13 (Year 11, Wave 3), attributable to the lack of any institutional support and motivation for participants post-schooling, together with the difficulty of obtaining accurate contact details 1 year in advance. A potential consequence of the low recruitment rate is self-selection bias, with more physically active girls being more motivated to participate in the study, resulting in a study sample which is not representative of the whole population of adolescent girls. A potential effect on longitudinal analyses of low or moderate retention rates is the possibility of further bias due to less physically active participants being more likely to drop out of the study. Also, it is possible that differences between the two cohorts might be confounded with the effects of a lower retention rate in the older cohort. Comparison of the baseline characteristics of those who completed all three surveys and those who did not [18] indicated a degree of self-selection bias towards girls with a greater competitive sport focus. Despite (and perhaps because of) these unavoidable limitations, therehas been little research in this domain, particularly longitudinal research, and so our findings, interpreted with appropriate caution, are important.

## Conclusion

In conclusion, while caution must be exercised because of the likely response bias towards those more physically active, the patterns of transition identified in this broadly-based study can be reasonably assumed to apply to adolescent girls throughout Australia, and potentially to other developed countries with similar sport and PA opportunities and cultures. Specifically, PA did not significantly decrease during adolescence, but it was evident that there were very distinct changes with respect to the context of participation. There was a clear trend over time of decreases in competitive sport, and more specifically club sport participation. This decrease in organised, structured competitive PA was substituted by participation in non-competitive PA, which was often unstructured. Broadly, many females are meeting the recommended levels of PA throughout adolescence, and this is a positive for their physical health. However we must also consider this issue more closely, as there are additional social and psychological health benefits assocated with sport participation [25], which these females may be missing out on as they move through to adulthood.

## Abbreviations

ABS, Australian Bureau of Statistics; IRSAD, Index of Relative Socio-economic Advantage and Disadvantage; LTMVPA, Leisure Time Moderate and Vigorous Physical Activity; MET, metabolic equivalent; MET-min, MET-weighted LTMVPA; MVPA, Moderate and Vigorous Physical Activity; PA, physical activity; PE, physical education; PDPAR, Previous Day Physical Activity Recall; SEIFA, Socio-economic Indices for Areas
